# Osteoblastic differentiation and changes in the redox state in pulp stem cells by laser treatment

**DOI:** 10.1007/s10103-024-04016-z

**Published:** 2024-03-06

**Authors:** Lina M. Escobar, Marggie Grajales, Zita Bendahan, Sully Jaimes, Paula Baldión

**Affiliations:** 1 Grupo de Investigaciones Básicas y Aplicadas en Odontología, IBAPO Facultad de Odontología, Universidad Nacional de Colombia, Carrera 30 No. 45-03, Bloque 210, 111321 Bogotá, Colombia; 2 Departamento de Salud Oral, Facultad de Odontología, Universidad Nacional de Colombia, Bogotá, Colombia; 3 Unidad de Manejo Integral de Malformaciones Craneofaciales UMIMC, Facultad de Odontología, Universidad El Bosque, Bogotá, Colombia

**Keywords:** Human dental pulp stem cells, Low-level laser therapy, Osteoblast differentiation, Photobiomodulation, Reactive oxygen species

## Abstract

The aim of this study was to determine the effect of low-level laser therapy (LLLT) on cell proliferation, mitochondrial membrane potential changes (∆Ψm), reactive oxygen species (ROS), and osteoblast differentiation of human dental pulp stem cells (hDPSCs). These cells were irradiated with 660- and 940-nm lasers for 5 s, 50 s, and 180 s. Cell proliferation was assessed using the resazurin assay, cell differentiation by RUNX2 and BMP2 expression, and the presence of calcification nodules using alizarin-red S staining. ROS was determined by the dichlorofluorescein-diacetate technique and changes in ∆Ψm by the tetramethylrhodamine-ester assay. Data were analyzed by a Student’s *t*-test and Mann–Whitney *U* test. The 940-nm wavelength for 5 and 50 s increased proliferation at 4 days postirradiation. After 8 days, a significant decrease in proliferation was observed in all groups. Calcification nodules were evident in all groups, with a greater staining intensity in cells treated with a 940-nm laser for 50 s, an effect that correlated with increased RUNX2 and BMP2 expression. ROS production and Δψm increased independently of irradiation time. In conclusion, photobiomodulation (PBM) with LLLT induced morphological changes and reduced cell proliferation rate, which was associated with osteoblastic differentiation and increased ROS and Δψm, independent of wavelength and time.

## Introduction

Low-level laser therapy (LLLT) is a treatment that utilizes focused light to stimulate a process known as photobiomodulation (PBM), which is “a non-thermal process involving endogenous chromophores that elicit a photophysical and photochemical reaction at various biological scales” [1]. This therapy uses nonionizing light sources, including lasers, light-emitting diodes, and broadband light in the visible (400–700 nm) and near-infrared (700–1100 nm) electromagnetic spectrum [2]. During PBM, photons penetrate cells and are absorbed by the cytochrome-c complex within the mitochondria. This triggers a biological cascade that increases cellular metabolism, which has beneficial outcomes such as pain relief, immunomodulation, wound healing, and tissue regeneration [3, 4].

Previous studies have demonstrated that PBM attenuates proliferation, survival, and differentiation in various cell types, which makes it a useful tool for achieving the objectives of tissue engineering, such as reconstructing or regenerating deteriorated or damaged tissues [5, 6]. Tissue reconstruction is achieved by the combination of scaffolds, stem cells, and inducing factors that act together to regenerate injured or missing tissues [7]. Stem cells are capable of self-renewal, and because of their undifferentiated nature, they can be stimulated to perform specific biological tasks and differentiate into several cell types [8]. Adult mesenchymal stem cells (MSCs) can be isolated from adipose tissue [9], bone marrow [10], periodontal ligaments [11], and human dental pulp stem cells (hDPSCs) [12]. Currently, the latter is of significant interest for tissue engineering because of the prior development of standardized extraction protocols, which facilitate collection and the fact that they can be obtained from disposable samples. Another advantage is their capacity for proliferation and differentiation into multiple phenotypes, such as osteoblasts, chondrocytes, and adipocytes [13–15].

The effects of PBM on viability, proliferation, and differentiation into osteogenic lineage of MSCs remain controversial. Although there are no clear conclusions, several studies have shown that PBM enhances MSCs viability and proliferation, particularly when red laser wavelengths are used [16–21]. However, other studies have found that laser treatment does not increase osteoblast proliferation and differentiation compared with control cells [22–24]. These discrepancies may result from differences in the methodologies used in the studies and the fact that it is unclear how the variation in irradiation parameters (e.g., wavelength, fluence, power density, emission mode, and application time) influence the biomodulatory effect of the laser on mesenchymal cells.

Because the underlying mechanism that regulates the increase in cell proliferation, differentiation, and migration is not yet precise, the use of LLLT remains controversial. Among the best-known proposed mechanisms of PBM is the action of cytochrome-c oxidase (CCO), an essential photoreceptor in mitochondria that contributes to the maintenance of the mitochondrial transmembrane potential and, consequently, influences ATP production. In addition, functional changes in the mitochondrial electron transport chain can generate reactive oxygen species (ROS) and changes in the mitochondrial membrane potential (∆Ψm), which affect ATP production [2–25]. ROS plays an important role in cell signaling, proliferation, cycle regulation, and protein synthesis [21, 26]; however, the specific role of ROS in the differentiation of MSCs toward PBM-treated osteoblasts is unknown.

The clinical effects of laser therapy depend on the correct selection of irradiation parameters, including wavelength, fluence, power density, and application time [2, 17]. Therefore, the purpose of this study was to determine and compare the effect on the proliferation and osteoblastic differentiation of hDPSCs cells treated with two types of lasers of different wavelengths and different exposure times. In addition, we also evaluated the changes in ∆Ψm and ROS’s participation in the osteoblastic differentiation of mesenchymal cells stimulated by low-level laser irradiation.

## Materials and methods

### Cellular model of human dental pulp stem cells

#### Isolation of human dental pulp stem cells

Human dental pulp stem cells (hDPSCs) extracted for therapeutic indication were isolated and characterized. Following the protocol established by Gronthos et al. [12] and modified by Baldion et al. [27], dental pulp explants were obtained from healthy premolar and third molar extractions from individuals between 18 and 20 years old. Teeth collection, handling, and disposal were carried out in accordance with ethical standards of national and international legislation, and all volunteers signed the informed consent form before surgery. Cells were obtained from two independent cultures from two donors with three replicates each (*n* = 6). Briefly, the teeth were decontaminated after extraction by immersing in 5% sodium hypochlorite for 5 s and sectioned with a high-speed piece to obtain complete pulp tissue. Explants were immersed in low-glucose Dulbecco’s modified Eagle’s culture medium (DMEM) (Gibco, Thermo Fisher Scientific, Bremen, Germany), supplemented with 10% fetal bovine serum (Hyclone, Thermo Fisher Scientific, Bremen, Germany) and antibiotics. A dissociation medium containing collagenase (3 mg/ml) (Sigma-Aldrich, St Louis, MO, USA) and dispase (4 mg/ml) (Gibco) was added and incubated for 16 h in an incubator containing an atmosphere humidified with 5% CO_2_ at 37 °C. The suspension was centrifuged, and the precipitate was resuspended in a culture medium and seeded in 25 cm^2^ flasks until reaching 80% confluence.

#### Phenotypic and functional characterization of hDPSCs

**Fig. 1:**
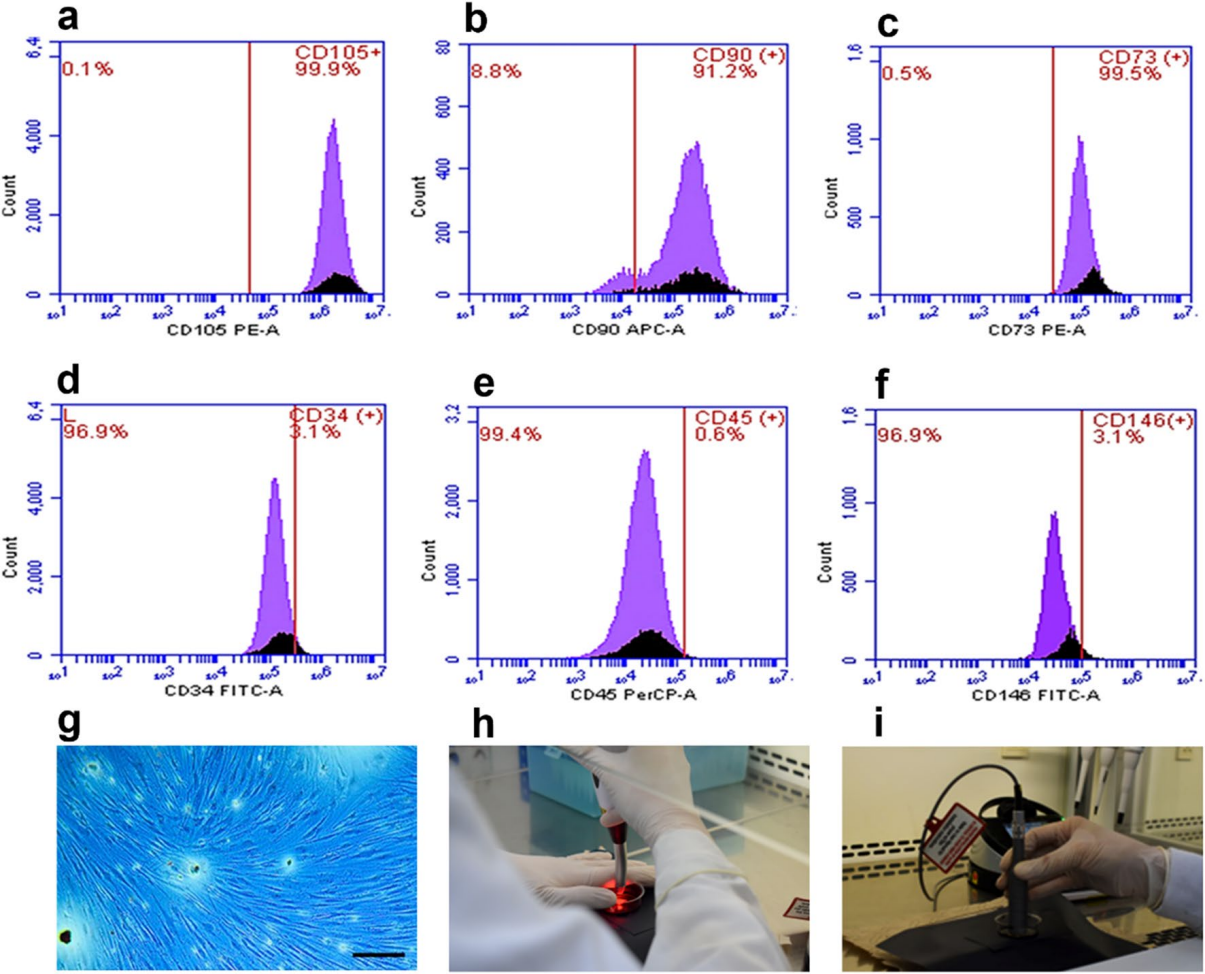
Isolation, characterization, and laser irradiation protocol for hDPSCs. Flow cytometry histograms with surface markers positive for CD105 (**a**), CD90 (**b**), and CD73 (**c**) and negative for CD34 (**d**), CD45 (**e**), and CD 146 (**f**). Isolated mesenchymal cells with fibroblastoid appearance (**g**). Magnification bar: 200 µm. Laser irradiation with Duo laser (MM Optics Ltda, Sao Paulo, Brazil) 660-nm wavelength (**h**) or Epic X laser (Biolase, CA, USA) 940-nm wavelength (**i**)

The characterization of hDPSCs was performed using criteria previously defined by the International Society for Cell Therapy [8]. The morphological characteristics of mesenchymal cells were evaluated by phase contrast light microscopy to corroborate cell adherence to plastic and the spindle-shaped appearance and abundant lysosomes [28]. Membrane markers were assessed by flow cytometry. The cultured cells were trypsinized, centrifuged, and resuspended in 100 µL of 1X phosphate-buffered saline (PBS). The cells were then incubated with 10 µL of monoclonal antibodies: PE Mouse Anti-Human CD34, PE Mouse Anti-Human CD45, APC Mouse Anti-Human CD73, FITC Mouse Anti-Human CD90, and PerCP-Cy™5.5 Mouse Anti-Human CD105 (BD Biosciences, San Jose, CA, USA). Cells without antibody labeling were used as a negative control. The analysis was performed on an Accuri C6 flow cytometer (BD Biosciences). A homogeneous population was characterized if cells were positive for CD73, CD90, and CD105, and negative for the early hematopoietic markers CD34 and CD45 [29] (Fig. 1). Finally, their differentiation capacity toward an osteoblast lineage was determined by culturing the cells in differentiation medium DMEM supplemented with 0.1 mM dexamethasone, 10 mM ß-glycerolphosphate, and 0.2 mM ascorbic acid. Cell differentiation was evaluated after 21 days of exposure to this medium [30].

### Treatment of hDPSCs cells with LLLT

The laser was applied to the cell cultures of the experimental groups using two diode lasers of different wavelengths, 660 nm (visible red) and 940 nm (near-infrared), with previous calibration of the equipment with an optical power meter (Nova II P/N 7Z01550, MKS Ophir, Jerusalem, Israel). The characteristics of the lasers and application parameters are based on the protocol for reporting application parameters for clinical and laboratory studies [31] (Tables 1 and 2). To determine the effect of LLLT, the cells were seeded into culture plates and divided into the following experimental groups: (i) control group, nonirradiated cells; (ii) irradiation with a 660 nm diode laser for 5 s; (iii) irradiation with a 660 nm diode laser for 50 s; (iv) irradiation with a 660 nm diode laser for 180 s; (v) irradiation with a 940 nm diode laser for 5 s; (vi) irradiation with a 940 nm diode laser for 50 s, and (vii) irradiation with a 940 nm diode laser for 180 s. The power used for the application was 0.1 W for both wavelengths. The doses applied to the cell cultures were calculated as square centimeters (cm^2^). The selection of application parameters was determined based on a previous systematic review [21]. A single application of the laser was performed for each experimental condition. The irradiation distance was calculated from the spot of the laser light beam to the bottom of the well without contacting the laser application tip inside the wells to avoid contamination (Fig. 1, Tables 1 and 2).

**Table 1:** Technical specifications of laser equipment

Wavelength (λ)	660 ± 10 nm	940 ± 10 nm Guide light 625–670 nm (laser diode)
Manufacturer	MM Optics Ltda	Biolase
Model identifier	Laser Duo	Epic X
Place of manufacture	SP–Brazil	California–USA
Emitter type	Diode GaAlAs laser	Diode InGaAs laser
*Laser beam delivery* system	Fiber optic	Fiber optic
Operating mode	Continuous-wave operation (CW operation) Settings: L1 mode	Continuous-wave operation (CW operation) Settings: pain therapy mode. Deep tissue handpiece—no spacer
*Laser beam profile*	Gaussian	Gaussian
Maximum output power	0,1 W ± 20%	10 W ± 20%

**Table 2:** Application parameters of the study

Wavelength	660 nm	940 nm
Beam spot size (cm^2^)	0.2	0.78
Area irradiated per application (cm^2^)	1	1
Area per well (96/12)* (cm^2^)	0.32/4	0.32/4
Power density (W/cm^2^)	0.5	0.13
Power output (W)	0.1	0.1
Exposure time (s)	5, 50, 180	5, 50, 180
Energy density (J/cm^2^)	2.5, 25, 90	0.64, 6.4, 23.1
Energy per point (J)	0.5, 5, 18	0.5, 5, 18
Total energy per well (96)* (J)	0.5, 5, 18	0.5, 5, 18
Total energy per well (12)* (J)	2, 20, 72	2, 20, 72
Application points per well (96)*	1	1
Application points per well (12)*	4	4
Application technique	Fixed-point with support	Fixed-point with support
Distance from laser spot (96/12*) (cm)	1.1/1.9	1.1/1.7
Number of applications	Single-dose	Single-dose

^*^Calculations performed for 96-well and 12-well cell culture plates, respectively

To determine cell viability, proliferation, and differentiation, cells were seeded into 12-well culture plates at a density of 5 × 10^3^ cells/well. To measure ROS production and mitochondrial membrane potential (Δψm), cells were seeded into 96-well culture plates at a density of 1 × 10^5^ cells per well. The lids of the culture plates and the culture medium were removed before irradiation. Once the irradiation was completed for each well, a fresh culture medium was immediately added. Both lasers were positioned with a stable handstand to ensure that the light was distributed perpendicular to each well in a homogeneous manner. The wells were covered with a black plastic sheet to avoid overlap and scattering during irradiation, leaving only the laser application area (1 cm^2^) uncovered. The divergence angle of the laser equipment was 8–22°, and the profile of the light beam had a Gaussian distribution, which is why the energy input may be slightly lower than calculated.

### Determination of viability, proliferation, and morphological changes of hDPSCs treated with LLLT

The viability and proliferation of hDPSCs with or without LLLT treatment were assessed by the trypan blue dye exclusion technique using a hemocytometer and the resazurin fluorometric assay.

Trypan blue is a stain used to quantify live cells by labeling dead cells exclusively. Live cells have an intact cell membrane and trypan blue cannot penetrate the cell membrane of live cells and enter the cytoplasm. In a dead cell, trypan blue passes through the porous cell membrane and enters the cytoplasm. Under light microscopy analysis, only dead cells have a blue color.

On the other hand, resazurin is a nonfluorescent compound that is irreversibly reduced to the highly fluorescent resofurin, and its increase is proportional to the mitochondrial activity of viable cells. Resazurin solution was added at 4.4 μM/per well to hDPSCs seeded into 12-well microplates at a density of 5 × 10^3^ cells/well and incubated at 37 °C for 4 h. Subsequently, the fluorescence intensity was measured using a microplate reader (Infinite M200 Pro, Tecan, Männedorf, Switzerland) at a wavelength of 535–595 nm [32].

### Evaluation of the osteoblastic differentiation of hDPSCs

hDPSCs were cultured in an osteogenic induction medium of DMEM supplemented with 100 U/ml penicillin, 100 µg/mL streptomycin, 0.1 μM dexamethasone, 5 mM β-glycerolphosphate, and 50 μg/mL ascorbic acid. The cells were treated with a differentiation medium for 7, 14, and 21 days in an incubator with a humidified atmosphere of 5% CO_2_ at 37 °C. The control group corresponded to cells maintained only in a culture medium without factors that induce osteoblastic differentiation.

#### In vitro* mineralization evaluation*

Cells were fixed with paraformaldehyde (PFA) and stained with 2% alizarin red S. Subsequently, the dye was extracted with 1X PBS, 10% acetic acid, and isopropanol solution for 16 h. The formation of calcification nodules was determined using an inverted microscope, and the dye’s absorbance was measured using a microplate reader (Infinite M200, Tecan) at 550 nm [33].

#### Osteogenic differentiation gene expression

Total RNA was extracted from untreated and treated hDPSCs cells using the Quick-RNA MicroPrep kit (Zymo Research, Irvine, USA) according to the manufacturer’s protocol. Subsequently, cDNA was synthesized by reverse transcription (RT) using the ProtoScript ll First Strand cDNA Synthesis kit (BIOHAUS SAS) following the manufacturer’s instructions. Real-time polymerase chain reaction (qRT-PCR) was performed using the Luna Universal kit (New England Biolabs, Ipswich, USA) to determine the expression of two important genes in the osteoblastic differentiation process: runt-related transcription factor 2 (*RUNX2*) and bone morphogenetic protein 2 (*BMP2*). A CFX96 Real-Time thermal cycler detection system (Bio-Rad; Hercules, USA) was used. The amplification conditions included 3 min at 95 °C and 50 cycles of 10 s at 95 °C, 30 s at 60 °C and 20 s at 72 °C. A final step of 5 s at 65 °C and 5 s at 95 °C followed. The primers used are listed in Table 3. The PCR efficiencies were calculated with the LinRegPCR program (Academic Medical Center, A.M.C., Amsterdam, Netherlands), and the relative quantification was determined following the Scheffe method using glyceraldehyde 3-phosphate dehydrogenase (GAPDH) gene as control [34, 35].

**Table 3:** Primers used in this study

Gen	Forward primer	Reverse primer	Amplicon size (bp)
*RUNX2*	5′-CATCTAATGACACCACCAGGC-3′	5′-GCCTACAAAGGTGGGTTTGA-3′	168
*BMP2*	5′-CGAAACACAAACAGCGGAAAC-3′	5′-GCCACATCCAGTCGTTCCA-3′	97
*GAPDH*	5′-GAAGGTGAAGGTCGGAGTC-3′	5′GAAGATGGTGATGGATTTC-3′	226

*RUNX2* runt-related transcription factor 2, *BMP2* bone morphogenic protein 2, *GAPDH* glyceraldehyde-3-phosphate dehydrogenase

### Evaluation of the intracellular redox state

#### Determination of reactive oxygen species (ROS) production in hDPSCs induced to osteoblast differentiation

ROS production induced by exposure to different laser wavelengths and exposure times was quantified by intracellular oxidation using 2′,7′-dichlorodihydrofluorescein diacetate at 25 uM (H2DCF-DA, Sigma-Aldrich; St Louis, MO, USA). In the presence of ROS, it is oxidized to a highly fluorescent compound, 2′,7′-dichlorofluorescein (DFC). hDPSCs were exposed to the H2DCF-DA probe for 1 h at 37 °C in the dark. Subsequently, the cells were washed and exposed to 1X TBHP (tert-Butyl H_2_O_2_) as a positive control for ROS generation and to 1X TBPH + 50 mg/ml AA (tert-Butyl H_2_O_2_ + ascorbic acid) as a ROS production inhibitor for 30 min following irradiation with LLLT [36–38]. Quantitative evaluation of the fluorescence signal was used to determine the change as a function of emitted intensity in unexposed cells versus treated cells using a ClarioStar Plus microplate reader fluorometer (B.M.G. Labtech, Germany) at a wavelength of 490 nm_exc_/520 nm_ems_. Simultaneously, the cells were seeded onto glass slides in 24-well plates and exposed to the same conditions as above to assess fluorescence using a Zeiss Axioimager A2 fluorescence microscope (Gottingen, Germany). The intracellular amount of ROS was directly proportional to the fluorescence intensity, and its increase was evaluated with respect to the unirradiated control cells. Three replicates were analyzed per condition.

#### Mitochondrial membrane potential assay

Mitochondrial membrane potential changes (Δψm) were assessed using the Tetramethyl rhodamine Ethyl Ester (TMRE) Assay Kit (Abcam, Cambridge, UK) following the manufacturer’s instructions. DPSCs (2.5 × 10^4^ cells/well) were seeded in dark 96-well plates and allowed to adhere for 20 h. After exposure to low-power laser irradiation at 660 nm and 940 nm for 5, 50, and 180 s, TMRE solution was added at a final concentration of 200 nM to each well and incubated for 30 min at 37 °C in the dark. During incubation, positively charged TMRE accumulates in the active mitochondria because of its high negative charge ratio. In contrast, in depolarized or inactive mitochondria, staining is not retained because of a collapse in membrane potential, which results in decreased fluorescence. Carbonyl cyanide-ptrifluoromethoxyphenylhydrazone (FCCP) at 20 µM was used as a positive depolarization control. It was added to the cells 10 min before staining with TMRE. The fluorescence intensity was determined using a spectrofluorometer (Infinite M200, Tecan) at a wavelength of 549 nm_exc_/575 nm_ems_ [36].

### Statistical analysis

Data were collected in Excel spreadsheets and exported to SPSS software, version 21.0 (SPSS, Chicago, IL, USA) for analysis. The data is presented as the mean and standard deviation. To analyze differences between groups of variables with parametric distribution, a Student’s *t*-test was used, and for those without parametric distribution, the Mann–Whitney *U* test was used. *P*-values ≤ 0.05 were considered statistically significant.

## Results

### Characterization of hDPSCs

Immunophenotyping revealed > 95% of the cells expressing the surface markers CD105 (Fig. 1a), CD90 (Fig. 1b), and CD73 (Fig. 1c). Low expression of the early hematopoietic cell markers CD34 (Fig. 1d), CD45 (Fig. 1e), and CD146 (Fig. 1f) was also observed. Morphological evaluation of hDPSCs obtained from dental pulp showed adherent monolayers with fibroblastic morphology (Fig. 1g).

### Effect of different LLLT application parameters on hDPSCs cell proliferation and morphology

**Fig. 2:**
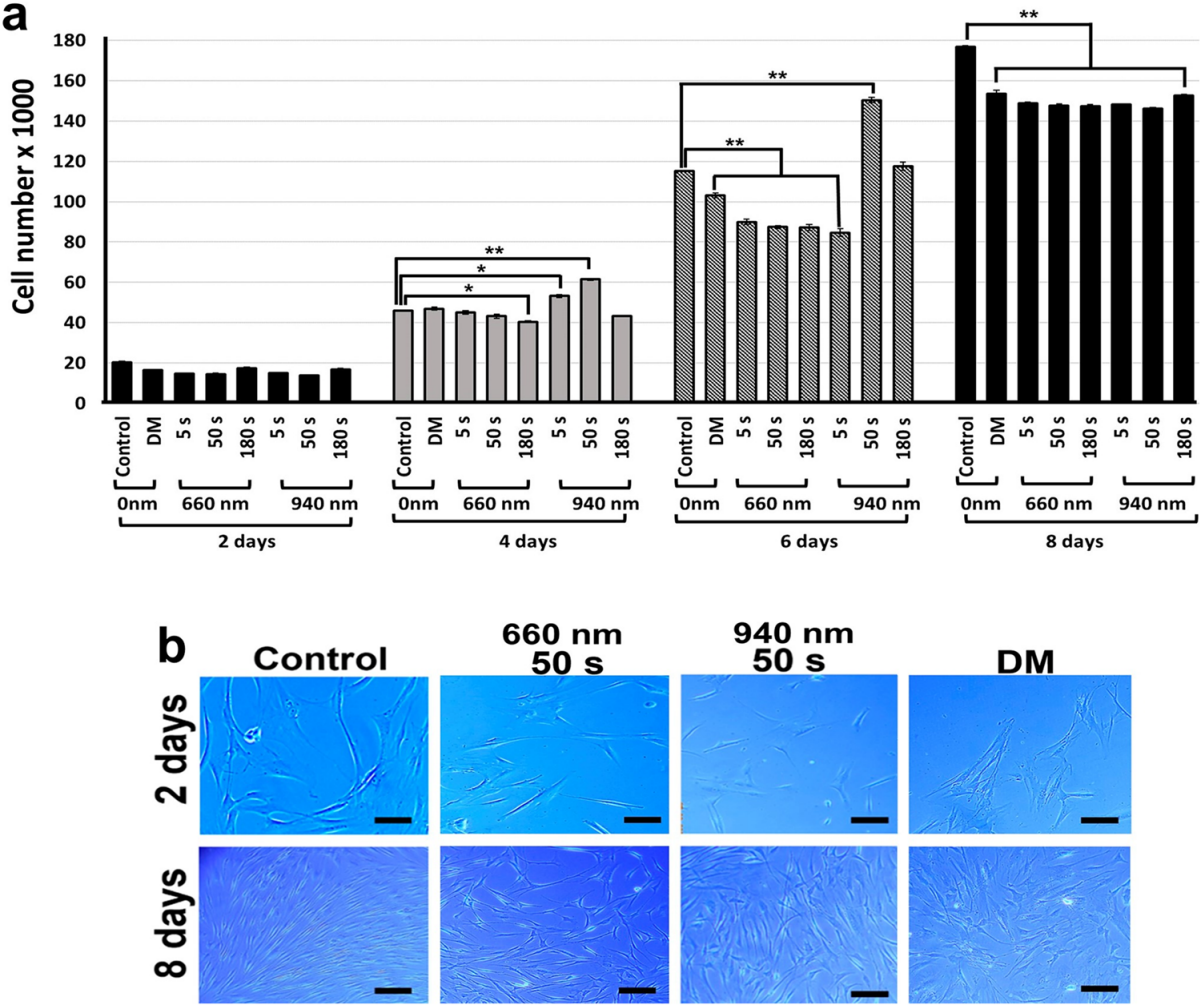
Changes in cell number and morphological analysis of hDPSCs cultures treated with LLLT. **a** Irradiation with a 660-nm laser for 5, 50, and 180 s caused a significant reduction in cell number at 6 days postirradiation. The 940-nm laser initially produced an increase in cell number when irradiating these cells for 5 and 50 s at 4 days postirradiation. At 8 days, all experimental groups exhibited a significant reduction in cell number compared with the untreated group (control). *p* < 0.05 (*), *p* < 0.01 (**). Data are expressed as averages ± DE differentiation medium (DM). **b** To determine morphologic changes, cells were treated with 660 nm and 940 nm LLLT for 5, 50, and 180 s and maintained in culture for 8 days postirradiation. The control group corresponded to hDPSCs without irradiation. Differentiation medium (DM). Magnification bar 200 µm

The hDPSCs cells irradiated with LLLT 660 nm for 180 s exhibited a significant reduction in proliferation from 4 days postirradiation, and this reduction was maintained over the 8 days of evaluation. In contrast, exposure to LLLT with the same wavelength for 5 and 50 s resulted in no changes in cell number at 4 days compared with the group of cells without laser treatment (control). It was only until the 6th and 8th day that a significant reduction in cell number was evident at these two irradiation times (Fig. 2a). Following irradiation at a 940-nm wavelength, a significant increase in cell number was observed in cultures irradiated for 5 and 50 s after 4 days. This increase was only maintained in the cultures irradiated with 940 nm for 50 s at 6 days postirradiation, as the group irradiated for 5 s had reduced cell numbers compared with the control group. When the cell number was analyzed after 8 days of LLLT, a significant reduction was evident in all cultures treated with the different wavelengths and irradiation times (20 to 25% reduction) and in cells treated with osteogenic differentiation medium (DM) (17% reduction) (Fig. 2a). The decrease in cell number was associated with a reduction in proliferation because no significant number of dead cells were observed when cell death was analyzed by the trypan blue exclusion assay (data not shown).

The morphological changes included more elongated cells with greater cellular prolongations and a lower cell density in the cultures irradiated with LLLT at the two wavelengths. These changes were more evident in cells treated with the longer wavelength (940 nm), regardless of the irradiation time. No monolayer detachment or cell death was evident in any of the experimental groups (Fig. 2b).

### Determination of hDPSCs differentiation toward osteoblastic lineage induced by LLLT

**Fig. 3:**
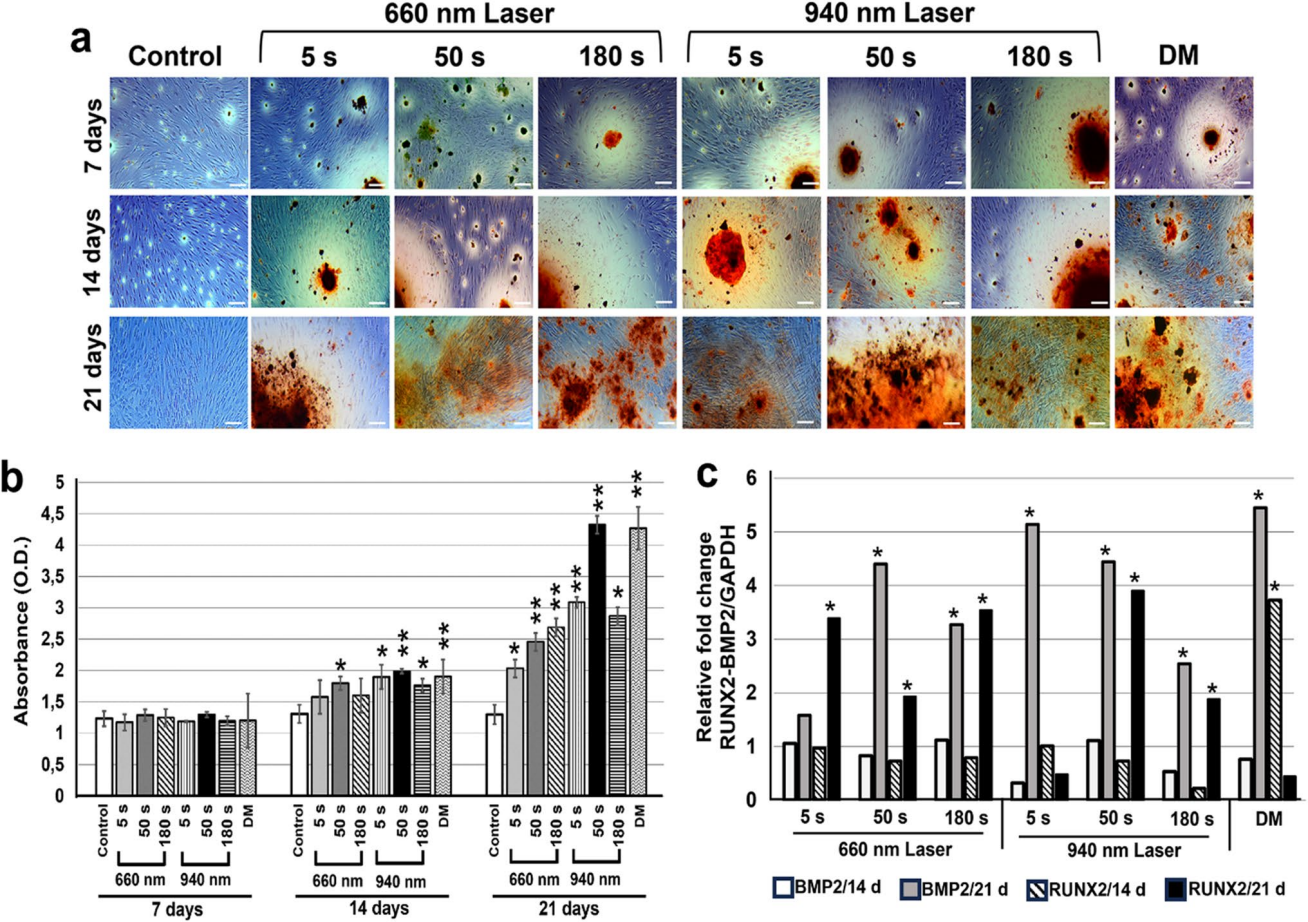
Mineralization of the extracellular matrix. **a** The mineralizing capacity of the extracellular matrix secreted by cells was determined by alizarin red staining. The strong intensity of the staining indicated the formation of calcification nodules. **b** Measured absorbance of alizarin red staining extracted from LLLT-treated cells at different wavelengths (660 nm and 940 nm) and times (5, 50, and 180 s) at 7-, 14-, and 21-days postirradiation. **c** Quantification of *RUNX2* and *BMP2* expression in 660-nm and 940-nm laser-treated hDPSCs for 14 and 21 days. Data are expressed relative to GAPDH gene expression levels and cells treated with osteogenic differentiation medium (DM) without laser irradiation were analyzed as a positive differentiation control. Cells without LLLT (control), differentiation medium (DM). *p* < 0.05 (*); *p* < 0.01 (**). Data are shown as the mean ± SD

To evaluate cell differentiation resulting from LLLT treatment, the formation of calcification nodules suggestive of osteoblastic differentiation was determined by microscopic analysis (Zeiss Axiovert 40 CFL microscope, Zeiss, Germany) and quantification of the extracted dye, alizarin red. No calcification nodules were evident in the control group at any measurement time (Fig. 3a and b). Cells treated with DM were used as a positive differentiation control.

At 7 days postirradiation, a few calcification nodules were formed in cultures irradiated with the two wavelengths and at different LLLT treatment exposure times (Fig. 3a and b). This mineralization process was increased at 14 and 21 days postirradiation under all experimental conditions compared with cultures without LLLT exposure (control). The formation of these nodules at 21 days postirradiation was significantly higher in cells treated with LLLT at 940 nm for 50 s, as observed by microscopy and the quantification of alizarin red (Fig. 3a and b). The values obtained in this experimental group were similar to those observed in the cells treated with DM for 21 days.

Changes in the expression of the two differentiation markers, *RUNX2* and *BMP2*, were determined by qRT-PCR analysis (Fig. 3c). Treatment of hDPSC with 660-nm laser for 50 s and 180 s caused an increase in both *RUNX2* and *BMP2* expression at 21 days postirradiation. Treatment with the 660-nm laser for 5 s increased *RUNX2* expression 3.5-fold at 21 days posttreatment, but no change in *BMP2* expression was observed at 21 days postirradiation. In addition, treating cells at a wavelength of 940 nm for 50 s also resulted in an increase in *BMP2* (4.5-fold) and *RUNX2* (3.8-fold) expression at 21 days postirradiation. Treating cells with the 940-nm laser for 180 s induced a 2.5-fold increase in *BMP2* and a 1.9-fold increase in *RUNX2* at 21 days postirradiation. In cells stimulated to undergo osteogenic differentiation with DM, an increase in *RUNX2* expression was observed at 14 days and *BMP2* expression at 21 days of culture (Fig. 3c). These results suggest that LLLT at the two wavelengths stimulated the differentiation of hDPSC toward an osteoblastic lineage because laser treatment induced a reduction in cell proliferation, morphological changes, increased formation of calcification nodules, and increased expression of the differentiation markers, *BMP2* and *RUNX2*.

### Effect of LLLT on the intracellular redox state

#### Production of reactive oxygen species

**Fig. 4:**
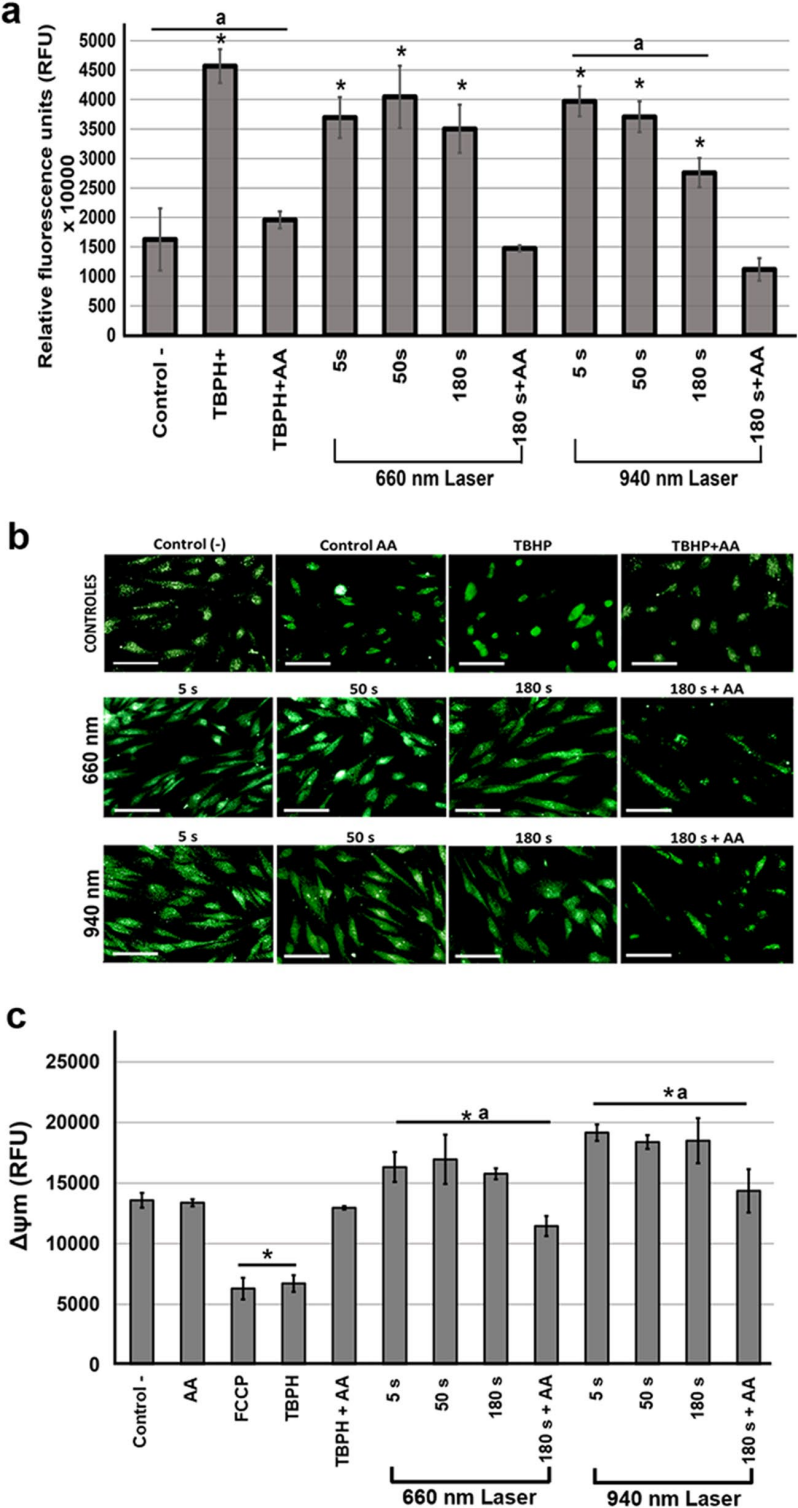
Effect of LLLT on ROS production and mitochondrial membrane potential in hDPSCs. **a** ROS production detected by the dichlorofluorescein-diacetate (DCF-DA) technique in cells irradiated with 660-nm and 940-nm lasers for 5, 50, and 180 s. Each treatment was compared in relative fluorescence units (RFU) and quantified by spectrofluorometry (485 nm_exc_/535 nm_ems_) to the negative control. ROS production corresponding to the positive control, tert-Butyl hydroperoxide (TBPH 1100X), and cells co-incubated with TBPH and 50 μg/mL ascorbic acid (AA), as well as ROS production by irradiation at both wavelengths during the maximum time evaluated (180 s) and AA are shown. **b** Changes in ROS production were recorded by Axiovert 40 CFL fluorescence microscopy (Carl Zeiss, USA) with a contrast of 5000, range of 1.05, brightness of 13,225, and an exposure time of 89.3 ms. Bar: 100 µm. **c** To evaluate changes in membrane potential, the cells were irradiated with 660-nm and 940-nm lasers for 5, 50, and 180 s. The fluorescence intensity obtained for each treatment was compared with the negative control (untreated cells), which was designated baseline mitochondrial membrane potential (Δψm). Fluorescence intensity corresponding to the positive control (TBHP) and cells irradiated for the longest exposure time (180 s) at each wavelength and co-incubated with 50 μg/mL ascorbic acid (AA) is shown. Fluorescence intensity was determined with a spectrofluorometer at a wavelength of 549 nm_exc_/575 nm_ems_. Results represent the mean ± SD of three independent experiments in triplicate (*n* = 9). Statistically significant differences between treated and untreated control groups are indicated by asterisks (*p* < 0.05). Lowercase letters (a) show significant differences between test groups (*p* < 0.05)

LLLT induces increased ROS in human dental pulp mesenchymal cells. The hDPSCs were exposed to 660-nm and 940-nm laser irradiation conditions to evaluate ROS production in the intracellular environment. A low level of ROS was detected in control cells with DMEM. In contrast, when cells were exposed to tert-Butyl hydroperoxide (TBHP) at 1100X, a significant increase of ROS was observed, which was reversed with 50 μg/mL ascorbic acid (AA). ROS production in hDPSCs following exposure to various laser irradiation conditions increased significantly, regardless of the wavelength used (*P* < 0.05). Using the 660-nm laser, no statistically significant difference was observed between 5, 50, and 180 s of irradiation (*P* > 0.05). In contrast, treatment with the 940-nm laser caused a tendency toward an inversely proportional behavior between irradiation time and ROS production, indicating lower ROS production in cells exposed for 180 s. The reduction of intracellular ROS by co-incubation with AA suggests a recovery of intracellular ROS levels by the antioxidant substance, which neutralizes the generated ROS (Fig. 4a).

Fluorescence microscopy corroborated the results in cells exposed to the laser at the two wavelengths as increased production of ROS (fluorescent green) was observed compared with control cells not exposed or treated with AA (Fig. 4b). These results indicated that the increase in intracellular ROS levels is one response of hDPSCs to LLLT exposure.

#### LLLT-induced increase in mitochondrial membrane potential in hDPSCs

To assess the mitochondrial response to laser irradiation, changes in the Δψm were determined using the tetramethylrhodamine methyl ester (TMRM) assay (Abcam, Cambridge, UK) with spectrofluorometry. In cells exposed to AA, no significant changes in Δψm were observed, respecting the untreated cell control. Carbonyl cyanide-ptrifluoromethoxyphenylhydrazone (FCCP) was used as a positive control for the uncoupling of oxidative phosphorylation as well as TBHP at 1100X as an oxidative stress induction control. Cells exposed to the positive controls exhibited a 46% (FCCP) and 49% (TBHP) decrease in Δψm due to the inability of mitochondria to retain TMRE. When evaluating the Δψm of TBHP/AA-treated cells, 41% of Δψm was observed in untreated cells (Fig. 4c). These data indicated that irradiation with both wavelengths induces an increase of Δψm regardless of irradiation time. The 940-nm wavelength showed a greater tendency to increase Δψm at all three exposure times compared with the control group and to irradiation with the 660-nm laser. No significant difference was observed between the three irradiation times with both wavelengths, 660 nm and 940 nm (*P* > 0.05).

## Discussion

In the present study was determined the effect of LLLT on proliferation, ROS production, changes in ∆Ψm, and osteoblastic differentiation of hDPSCs treated with two wavelengths (660 nm and 940 nm) during different exposure times (5 s, 50 s, and 180 s). When evaluating postirradiation effects, a decrease in cell proliferation was observed 8 days after irradiation, irrespective of wavelength and irradiation time. This may be related to increased differentiation toward the osteoblastic lineage, as evidenced by the expression of RUNX2 and BMP2, which increased 21 days after treatment in all experimental groups. Similarly, a significant increase in the presence of calcification nodules was observed mainly in the group exposed to the 940-nm laser at 50 s, which also exhibited the highest values of alizarin red S. Additionally, an increase in ROS and ΔΨm levels was evident, which has previously been reported as a possible effect associated with PBM [2, 22, 39].

It was previously reported that the effect of LLLT, both in vivo and in vitro, depends on the application parameters used [40]. The treatment will likely be ineffective if the wrong parameters are applied [41]. Several studies [3, 42] evaluating different doses of LLLT have reported a biphasic response, and the Arndt–Schulz law is accepted as a suitable model to describe the effects of this therapy. According to this law, when energy is insufficient, there is no response (because the minimum threshold has not been reached). If more energy is applied, the threshold is exceeded, and biostimulation is achieved; however, when energy is excessive, stimulation disappears and is replaced by bio inhibition [43]. Therefore, it is essential to determine, through scientific studies, the precise and appropriate parameters of low-level laser irradiation to achieve the desired objectives in a reproducible manner.

The criteria for selecting the application parameters in this study were based on a previous systematic review that analyzed dosimetry during in vitro studies [21] and the range of established irradiation parameters (wavelength, power, power density, energy density, application time) in which PBM was reported to be effective [44], considering the upper and lower limits of these parameters. Since we intended to evaluate wavelengths belonging to the red and near-infrared electromagnetic spectrum and according to previous literature reports, the two wavelengths selected in the present study were 660 nm and 940 nm (Table 2). They belonged to the red and near-infrared range of the electromagnetic spectrum. This allowed us to observe the behavior of LLLT in hDPSC cultures.

Systematic reviews [16, 19] indicated that 660 nm red laser had been used in most of the included studies. Another study reported using 810 nm and 980 nm diode lasers, with output power ranging from 20 to 100 mW. This is consistent with a more recent systematic review [21], which indicated that 13 studies used diode lasers with wavelengths between 635 and 980 nm. Only one study used an Nd: YAG laser (λ1064 nm). Energy densities ranged from 0.378 to 78.75 J/cm^2^ and irradiation times were between 1.5 and 300 s. All these reviews concluded that there is a positive effect of LLLT on cell proliferation and differentiation; however, the evidence was weak due to the heterogeneity of the methods used and the moderate risk of bias in the various studies.

In the present study, no significant changes in proliferation were observed in the short term (2nd day); however, by the 6th day, a decrease in proliferation was observed in the group exposed at 660 nm, as well as an increase in proliferation in the group exposed at 940 nm 50 s, which was subsequently reduced by the 8th day, when both wavelengths caused a significant decrease in proliferation compared to the control group, with no apparent reduction in cell viability. Furthermore, this reduction in cell number correlated with morphological changes, increased formation of calcification nodules, and expression of osteoblast differentiation marker genes, suggesting that both 660-nm and 940-nm lasers induced differentiation of hDPSCs toward an osteoblastic lineage.

Previous studies have demonstrated a differentiation-inducing effect in cells exposed to LLLT. Miglario et al. [39] used a 980-nm diode laser with irradiation times of 1, 5, 10, 25, and 50 s and energy fluences of 1.57, 7.87, 15.74, 39.37, and 78.75 J/cm^2^, to evaluate the effect on murine MC3T3-E1 preosteoblasts. Increased proliferation was observed when treated with 7.87 J/cm^2^ but decreased with 78.75 J/cm^2^ during 24 and 48 h following irradiation, suggesting that higher energy densities may have an inhibitory effect on the proliferation of this cell type. This agrees with the results presented in this study.

A study using a 635 nm diode laser at 30 mW/cm^2^ for 0, 34, 67, or 102 s (0, 1, 2, or 3 J/cm^2^) applied to MC3T3-E1 cells revealed that LLLT induced osteoblast differentiation into primary MSCs and osteoblast precursor cells, as evidenced by functional assays (calcium deposition) and expression of differentiation markers, including alkaline phosphatase (ALP), osteocalcin and BMP2. Furthermore, the group that showed a significant increase in BMP2 expression was those treated with 635 nm at 3 J/cm^2^ [45]. Another study exposed Saos-2 osteoblasts to three different PBM treatments, including 635 ± 5 nm, 808 ± 10 nm, and 405 nm. Compared to controls, a statistically significant increase in RUNX2 and ALP expression was observed after 635-nm laser PBM. Treatment with 808 nm increased the expression of RUNX2 but not ALP. No variations in the expression of these genes were detected in cells subjected to PBM at 405 nm compared to control cells [2].

When studying the effect of 808-nm laser on human periodontal ligament stem cells, a significant increase in cell proliferation and osteogenic differentiation (through the expression of RUNX2, Col-1, ALP, and osteonectin) was observed, especially at 1 and 2 J/cm^2^ combined with vitamin D [46]. Similarly, in another study [47] comparing the effect of a single and double dose 808-nm laser with a control group in DPSC, the double dose irradiation groups were consistent with an increase in calcium and ALP formation compared to the single irradiation group. In addition, osteopontin expression was significantly increased in the double-dose group compared to the single-dose group. Positive staining with alizarin red S and ALP confirmed the presence of calcium deposits in the analyzed samples. The above reports agreed with the results of increased RUNX2 expression 21 days after irradiation, especially in the 660 nm (red laser) at 5 s, 50 s, and 180 s, and 940 nm (near-infrared laser) groups at 50 s and 180 s, with PBM treatment at 940 nm for 50 s inducing the highest expression. In cells treated with 940 nm for 5 s, a lower expression of RUNX2 was observed compared to the other groups, which can be attributed to a low fluence applied (0.64 J/cm^2^), which was not sufficient to produce an effect on RUNX2 expression. As for BMP2 expression, higher expression was observed at 21 days in the 660 nm groups with application times of 50 and 180 s and in the 940 nm groups at 50 and 180 s. The group treated with 940 nm for 50 s exhibited the highest gene expression (4.5-fold).

Regarding the appearance of calcification nodules, the 940-nm laser group for 50 s (6.4 J/cm^2^) presented the highest alizarin red values, similar to the results reported by Sivakumar et al. [47], when applying a laser with a double dose of irradiation. However, in contrast, we found a significant increase in calcification nodule formation with a single irradiation dose.

Regarding intracellular redox state, when evaluating the relationship between ROS production and Δψm with low-level laser irradiation, an increase in Δψm was observed, which corresponded to an accumulation of intracellular ROS after irradiation with the two lasers (660 nm and 940 nm). The use of DCFH2-DA as a fluorescent probe to detect the redox state was useful for the identification of oxidative species, such as peroxides, superoxides, and nitric oxide [48], generated by laser PBM. ROS produced by the application of low-power lasers is presumably derived from endogenous sources, such as mitochondria, which are sensitive to red and near-infrared light. The effect of LLLT on metabolic activity is associated with the ability to stimulate electron transfer during oxidative phosphorylation, which promotes ATP production [25, 49]. This, combined with the results obtained from stimulation of the mitochondrial membrane potential, suggests an effect of LLLT on the inner mitochondrial membrane or mitochondrial protein complexes. The result is efficient electron transfer and adequate proton flux to catalyze ATP synthesis through activation of the transmembrane enzyme ATP synthase. Specifically, increased ATP synthesis is associated with light absorption by unit IV of the mitochondrial respiratory chain, which contains the chromophore CCO [50]. Consistent with these results, previous studies have indicated that ROS levels and ΔΨm are directly proportional. The rate of ROS production increases significantly when ΔΨm is above 140 mV and decreases by approximately 70% when the ΔΨm level drops to 10 mV [51]. This effect resulting from laser PBM is opposite to that obtained by exposure to TBPH, a prooxidant agent that induces excessive ROS production leading to a concomitant decrease in ΔΨm. This phenomenon has been termed “ROS-induced R.O.S. release,” a cycle of mitochondrial ROS formation and release through the mitochondrial permeability transition pore (mPTP). During prolonged oxidative stress, sustained opening of the mPTP leads to increased ROS production that causes cellular injury associated with decreased ΔΨm and ultimately affects cell survival, migration, proliferation, and differentiation.

Consequently, mitochondria are known to play an essential role in the regulation of apoptosis. The results showed that the Δψm of treated cells increased compared with TBPH-treated cells and even with untreated cells. Since Δψm decreases during apoptosis, LLLT may have a positive effect on reducing apoptosis, in part, through the PI3K/Akt signaling pathway. The increase in ROS because of LLLT exposure agrees with results reported by other authors [49, 52], who have described the beneficial effects of ROS accumulation at safe concentrations, in addition to improving total cellular antioxidant capacity and shifting the oxidative/antioxidative balance toward the antioxidant state.

## Conclusions

LLLT caused a reduction in the number of hDPSCs 8 days after irradiation. It also induced morphological changes, the formation of calcification nodules, and an increased expression of *RUNX2* and *BMP2*, suggesting the induction of hDPSCs differentiation toward the osteoblastic lineage. These changes were associated with an intracellular ROS and Δψm increase, regardless of wavelength and exposure time.

## Data Availability

The dataset supporting the conclusions of this article is available upon request to the corresponding author.
